# The use of saliva and blood progesterone to profile the menstrual cycles of youth professional football players

**DOI:** 10.3389/fspor.2024.1430158

**Published:** 2024-08-21

**Authors:** Eva Ferrer, Gil Rodas, Gregori Casals, Antoni Trilla, Laura Balagué-Dobon, Juan R. González, Katherine Ridley, Richard White, Richard J. Burden

**Affiliations:** ^1^ Sports Medicine Unit, Hospital Clinic and Sant Joan de Déu, Barcelona, Spain; ^2^ Barça Innovation Hub, Health & Wellness Area, Barcelona, Spain; ^3^ Medical Department of Futbol Club Barcelona, FIFA Medical Center of Excellence, Barcelona, Spain; ^4^ Faculty of Medicine and Health Sciences, University of Barcelona, Barcelona, Spain; ^5^ Department of Internal Medicine, Hospital Clinic of Barcelona, Barcelona, Spain; ^6^ Infectious Diseases Department, Hospital Clinic de Barcelona, Barcelona, Spain; ^7^ Barcelona Institute for Global Health (ISGlobal), Barcelona, Spain; ^8^ CIBER en Epidemiología (CIBERESP), Barcelona, Spain; ^9^ Health and Wellness Department, Mint Diagnostics, Sittingbourne, Kent, United Kingdom; ^10^ Health and Wellness Department, Sport in Balance, Essex, United Kingdom; ^11^ Female Athlete Health & Performance Department, UK Sports Institute, Manchester, United Kingdom; ^12^ Department of Sport and Exercise Sciences, Institute of Sport, Manchester Metropolitan University Institute of Sport, Manchester, United Kingdom

**Keywords:** female football, ovarian hormones, saliva progesterone, menstrual cycle, non-invasive method, capillary blood progesterone

## Abstract

**Background:**

Understanding individual ovarian hormone cycles and their relationship with health, performance and injuries is highly important to practitioners supporting female athletes. Venous blood sampling is the current gold standard for measuring the ovarian hormones, but the invasive nature of this method presents a major barrier in sport environments. Saliva analysis may offer an alternative method as it is non-invasive, allowing the sample to be collected “*in situ*”, with relative ease, necessary in applied sport environments.

**Objective:**

The aims of this study were: (i) To compare the concentration of progesterone between capillary blood and saliva, (ii) To assess the efficacy of weekly measurements of progesterone for determining if ovulation has occurred in elite eumenorrheic football players, and (iii) To establish a saliva criteria cut-off for establishing ovulation and assessing the sensitivity, specificity and accuracy values of the method.

**Methodology:**

Twenty-one professional and semi-professional, Spanish league female football players (18.6 ± 1.5 years, 58.1 ± 6.0 kg, 164.0 ± 4.8 cm) with natural menstrual cycles, completed the study. Capillary blood and saliva samples were collected from each participant on twelve occasions each separated by at least 7 days. All samples were collected in the morning, following an overnight fast.

**Results:**

According to luteal phase serum progesterone concentrations, 11 out of 21 (52%) players presented with menstrual irregularities (oligomenorrheic *n* = 6, anovulatory *n* = 4, amenorrhoeic *n* = 1). A significant correlation was observed between plasma and saliva progesterone in the estimated eumenorrheic group (*r* = 0.80, *p* = <0.001, 95% CI 0.72–0.86). The association between serum and saliva progesterone was weaker in the oligomenorrheic group (*r* = 0.47, *p* = <0.001, 95% CI 0.27–0.64) and was not present in the anovulatory or amenorrhoeic groups.

**Conclusions:**

Salivary measurements of progesterone are well correlated with capillary blood when taken during eumenorrheic menstrual cycles and presents a viable, non-invasive method of establishing characteristic progesterone fluctuations in applied sport settings. The strength of the association appears to be concentration dependent. A luteal phase saliva progesterone (P4) >50 pg/ml and >1.5× follicular baseline has good sensitivity, specificity, and accuracy to indicate ovulation compared to established criteria for serum progesterone.

## 1 Introduction

A higher incidence of menstrual irregularities has been reported in athletes compared to the general population, particularly at the professional level (1). Amenorrhea, oligomenorrhea and hypermenorrhea can range from 0% to 61% (2). For this reason, there is considerable interest in increasing the knowledge of the menstrual cycle (MC) physiology and its influence in sport performance and injury risk (3). Despite a current lack of evidence to support MC phase-based training interventions (4), menstrual cycle monitoring is becoming a more commonly used tool to help understand an individual's menstrual status and inform female athlete health and performance support (5). In addition, increased awareness of musculoskeletal injuries in female athletes has resulted in greater focus and interest on potential risk factors including the influence of MC phases (6, 7) and therefore the individual hormonal profile.

Effective MC monitoring requires objective measurement of ovarian hormones, notably oestrogen and progesterone, that fluctuate throughout the natural MC with high intra and inter-individual variability (8–10). Measurement of the ovarian hormones in venous blood is generally considered the gold standard, but it is invasive and often impractical (8). Capillary blood sampling offers a less invasive alternative, yet still requires qualified professionals to carry it out, limiting its feasibility in many elite sport environments.

The characteristic fluctuations of progesterone during a natural MC have previously been observed in saliva (11–13). Lipid-soluble unconjugated progesterone enters saliva predominantly via the intracellular route, and concentrations are independent of saliva flow rate, and closely approximate their unbound concentration in plasma (14). Thus, salivary progesterone can be used as part of an assessment of normal ovarian function (15, 16).

Measurement of salivary progesterone could provide a reliable reference for its blood concentration (17) and accessible method for capturing the key characteristics of a eumenorrheic MC, thereby enabling greater exploration of the influence of the MC on health, exercise performance and injury risk. For this reason, our aims for this study were (1) to compare measurements of progesterone between capillary blood and saliva within a group of elite football players, and (2) assess the efficacy of a protocol to indirectly determine ovulation by weekly progesterone measurement and (3) to establish a saliva criteria cut-off for the sensitivity, specificity and accuracy values of the method.

## 2 Material and methods

### 2.1 Design

On-field longitudinal study based on repeated measures of an elite football cohort.

### 2.2 Subjects

Twenty-one professional and semi-professional female football players were recruited for the study. All players monitored their menstrual cycle, using a calendar-based method (club app) and reported as having natural menstrual cycle (no use of hormonal contraception for at least 6 months, no switch to hormonal contraception during the study, not taking hormone-based medication), and provided informed consent. Ethical approval was granted by Clinical Research of the Catalan Sports Council Ethical Review Committee (application number: 012/CEICGC/2021) and a local Barça Innovation Hub Ethical Committee.

### 2.3 Sample collection

Capillary blood and saliva samples were collected from the players 1 day per week, for 12 weeks which corresponds approximately to three individual menstrual cycles. All players provided samples on the same day irrespective of individual menstrual cycle phase. All capillary blood and saliva samples were collected within 5–15 min of each other, in the morning with the players in a rested and fasted state.

*Capillary blood* was collected from a finger using specific Microvette capillary tubes (Sarstedt, Germany) by a healthcare professional for subsequent analysis. Samples were centrifuged (3,000 g × 10 min) and plasma concentration levels of progesterone were measured by a chemiluminescence immunoassay performed on an Atellica IM 1,600 instrument (Siemens Healthineers, Germany) (18). Measuring interval was 0.21–60 ng/ml and assay imprecision was <10%.

*Saliva samples* were self-collected by each player, using the passive drool method, where 1.5 ml of saliva was collected into Eppendorf tubes (Germany) and visually inspected for blood contamination; and although samples can be stable at room temperature for a week or longer (19) these were immediately frozen at −20°C at the Club's medical facilities before subsequent laboratory analysis (Mint Diagnostics, Kent, UK). Prior to collection of saliva, participants refrained from eating, drinking, or brushing teeth for 30 min to ensure sample clarity and viability. Once at the laboratory, samples were centrifuged (10 min at 2,000–3,000 g at room temperature). Samples were tested in duplicate to determine progesterone concentrations (average CV 2.2%) using commercially available enzyme immunoassays (IBL International, Germany). Each sample was pipetted into the well of the Microtiter Plate. Samples were then incubated for 60 min at room temperature (18–25°C). Enzyme Conjugate was pipetted into the well and mixed for 10 s using a plate shaker. Samples were then covered with adhesive foil and incubated for a further 60 min at room temperature (18–25°C). The foil was removed, and the incubation solution discarded. Tetramethylbenzidine (TMB) substrate solution was pipetted into each well and incubated for 30 min at room temperature (18–25°C). The substrate reaction was stopped by adding TMB stop solution into each well, and the contents mixed by gently shaking the plate—the color then changed from blue to yellow. A photometer Chromate 4,300 (Awareness Technology Inc, USA) was used to measure the optical density at 450 nm (reference wavelength: 600–650 nm) within 15 min after pipetting of the stop solution. Measuring range was 25–5,000 pg/ml and assay imprecision was <10%.

### 2.4 Determining ovulation

Following Wideman et al., a menstrual cycle was identified as being ovulatory, when luteal capillary blood progesterone levels exceeded 2 ng/ml (20).

### 2.5 Blood and saliva sample correlation analysis

Statistical analysis of the by-group saliva and capillary blood concentrations were carried out using the Python data science stack (NumPy, Pandas, SciPy). Pearson's correlation coefficient was employed in the individual and group saliva-blood correlations. We conducted a retrospective analysis of menstrual cycle data to evaluate ovulation status based on saliva progesterone (saliva P4) values only. Menstrual cycle lengths were calculated for each participant by counting from the first day of menses up to and including the day before the next menses. For each MC, we extracted the maximum luteal saliva P4 (*P4_max_*) from measurements within the second half of the individual's cycle. We also calculated the median follicular saliva P4 (P4_follicular_) from the first half of the cycle. The ratio of *P4_max_: P4­_follicular_* was computed as *P4_ratio_*. Based on cycle classification using blood criteria (receiver operating characteristic (ROC) curve analysis (Scikit-learn) (21) was applied to the set of complete cycles to provide thresholds for P4_ratio_ and P4_max_ as predictors of ovulation.

## 3 Results

### 3.1 Subjects

Twenty-one female Caucasian players (Age, 18.6 ± 1.5 years, body mass 58.1 ± 6.0 kg, height 164.0 ± 4.8 cm) were classified as assumed ovulatory (*n* = 10), oligomenorrheic (*n *= 6), anovulatory (*n* = 4) or amenorrhoeic (*n* = 1) according to specific criteria: presence of menses, MC length 21–35 days, luteal phase blood P4 > 2 ng/ml (20) (Table 1).

**Table 1:** Group classification according to different menstrual cycle.

	Assumed Ovulatory	Oligomenorrheic	Anovulatory	Amenorrhoeic
*n*	10	6	4	1
Age (years)	18.6 ± 1.6	18.5 ± 1.4	18.5 ± 2.1	19
Menarche (years)	12.7 ± 0.8	12.5 ± 1.0	12.8 ± 1.0	13

### 3.2 Capillary blood & saliva comparison

Based on the blood criteria (luteal phase blood P4 > 2 ng/ml), 52% of the players monitored presented with assumed menstrual irregularities. There were no differences between groups in age (*p* > 0.05) or age of menarche (*p* > 0.05).

A strong and significant correlation between progesterone levels measured in plasma and saliva was observed in the eumenorrheic group (*r* = 0.80, *p* < 0.001, 95% CI 0.72–0.86).

A moderate and significant correlation was found in the oligomenorrheic group (*r* = 0.47, *p* < 0.001, 95% CI 0.27–0.64). There were no significant correlations found in the anovulatory (*r* = 0.10, *p* = 0.947, 95% CI −0.28 to −0.30) or amenorrhoeic (*r* = −0.38, *p* = 0.19, 95% CI −0.75 to −0.21) group.

**Figure 1:**
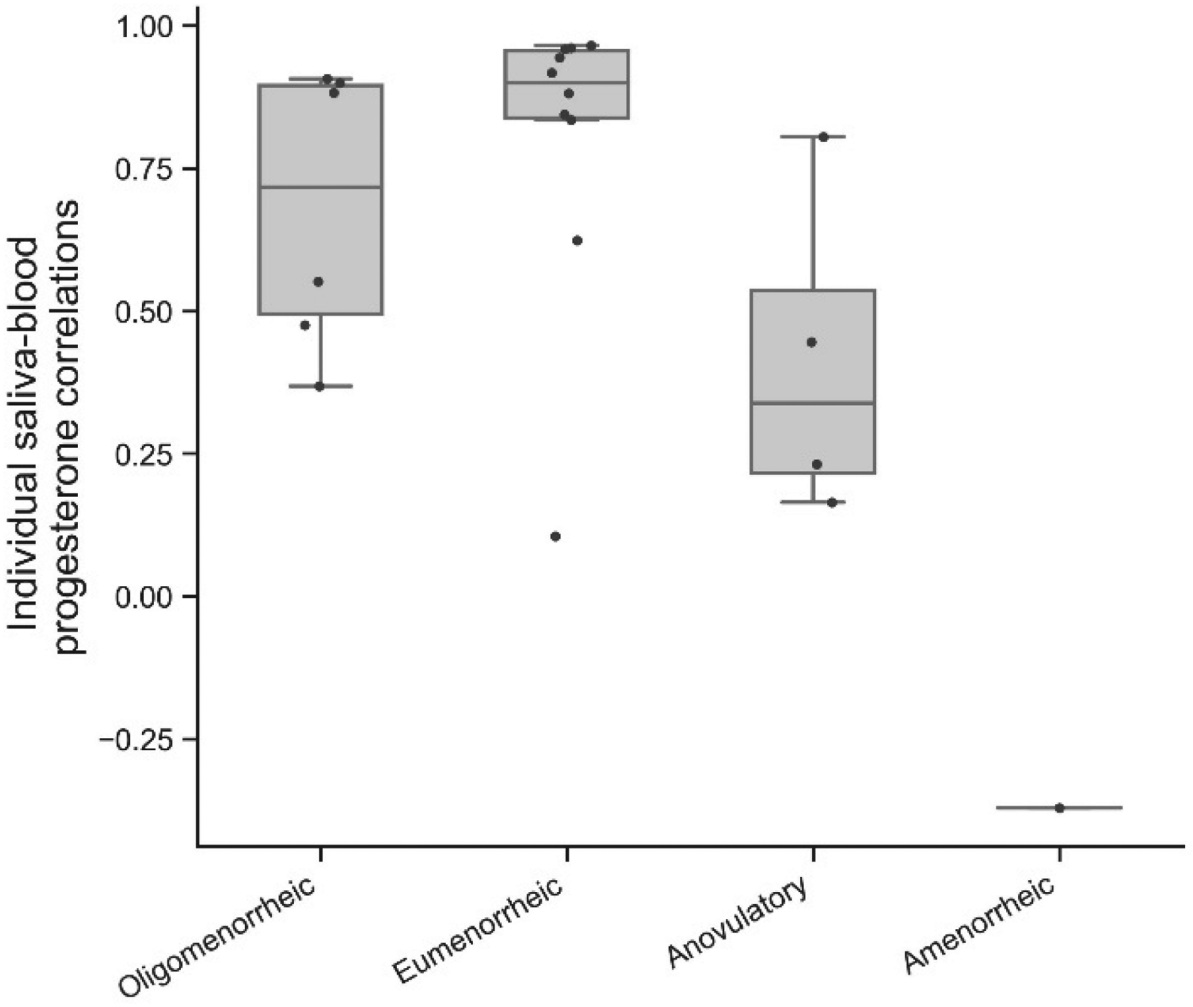
Individual correlations between capillary blood and salivary progesterone concentration, classified by menstrual status**.**

Saliva and capillary blood correlations for individual players are presented in Figure 1. Median and interquartile range for each group were eumenorrheic *r* = 0.90 (0.84–0.96), oligomenorrheic *r* = 0.71 (0.49–0.90) and anovulatory *r* = 0.34 (0.22–0.54).

### 3.3 Ovulation determination

To test the efficacy of using weekly salivary progesterone measurements to estimate the occurrence of ovulation, the sensitivity, specificity, and accuracy of luteal phase salivary progesterone was assessed against established blood-based criteria for ovulation (20).

We identified that a luteal phase salivary progesterone level being greater than 1.85 times the follicular baseline demonstrated good sensitivity (0.85), specificity (0.91), and accuracy (0.88) in indicating ovulation when compared to established criteria for blood progesterone. Using these criteria, we classified ten players as eumenorrheic, six as oligomenorrheic, four as anovulatory and one player as amenorrhoeic (Table 1).

### 3.4 Saliva criteria cut-off

Fifty complete menstrual cycles were used to evaluate different criteria for predicting ovulation including 24 ovulatory cycles and 26 anovulatory cycles according to the blood criteria. Receiver Operating Characteristic (ROC) curve analysis of *P4_ratio_* as a predictive variable identified 1.85 as the optimal threshold for balancing sensitivity and specificity with an area under the curve (AUROC) of 0.86. Separately, ROC curve analysis of P4_max_ yielded an AUROC of 0.85 with an optimal threshold of 76 pg/ml.

The sensitivity (true positive rate), specificity (true negative rate) and overall accuracy of using both P4_ratio_ and P4_max_ thresholds individually and combining the two are shown in Table 2.

**Table 2:** Saliva criteria cut-off.

Threshold set	P4_ratio_ threshold	P4_max_ threshold	Sensitivity	Specificity	Accuracy
1	1.85	–	85%	91%	88%
2	–	76	78%	96%	86%
3	1.85	76	74%	100%	86%

## 4 Discussion

To the knowledge of the authors, this is the first study examining the utility of capillary and salivary measures of progesterone taken in an elite training environment to understand the menstrual health of female professional football players. The aim of our study was to (i) to compare the concentration of progesterone between capillary blood and saliva, (ii) assess the efficacy of weekly measurements of progesterone for estimating if ovulation has occurred in elite football players, and (iii) to establish threshold criteria for salivary progesterone and assess the sensitivity, specificity, and accuracy of the method. The main finding of this study is that salivary measurements of progesterone are well correlated with capillary blood when taken during assumed-ovulatory MCs.

### 4.1 Saliva samples compared with capillary blood samples

Salivary progesterone represents the biologically active fraction of the hormone found in blood i.e., not bound to sex hormone-binding globulin (22) represents approximately 1%–2% of the total serum concentration of the hormone (23). A primary finding of this study is that salivary measurements of progesterone are well correlated with capillary blood when sampled from individuals whose MCs are assumed to be ovulatory due to luteal concentrations of progesterone. This agrees with previous studies exploring the association between serum and salivary measures of progesterone, showing strong correlations in healthy women with regular menstrual cycles (24) and women undergoing assisted reproduction treatment (25, 26).

**Figure 2:**
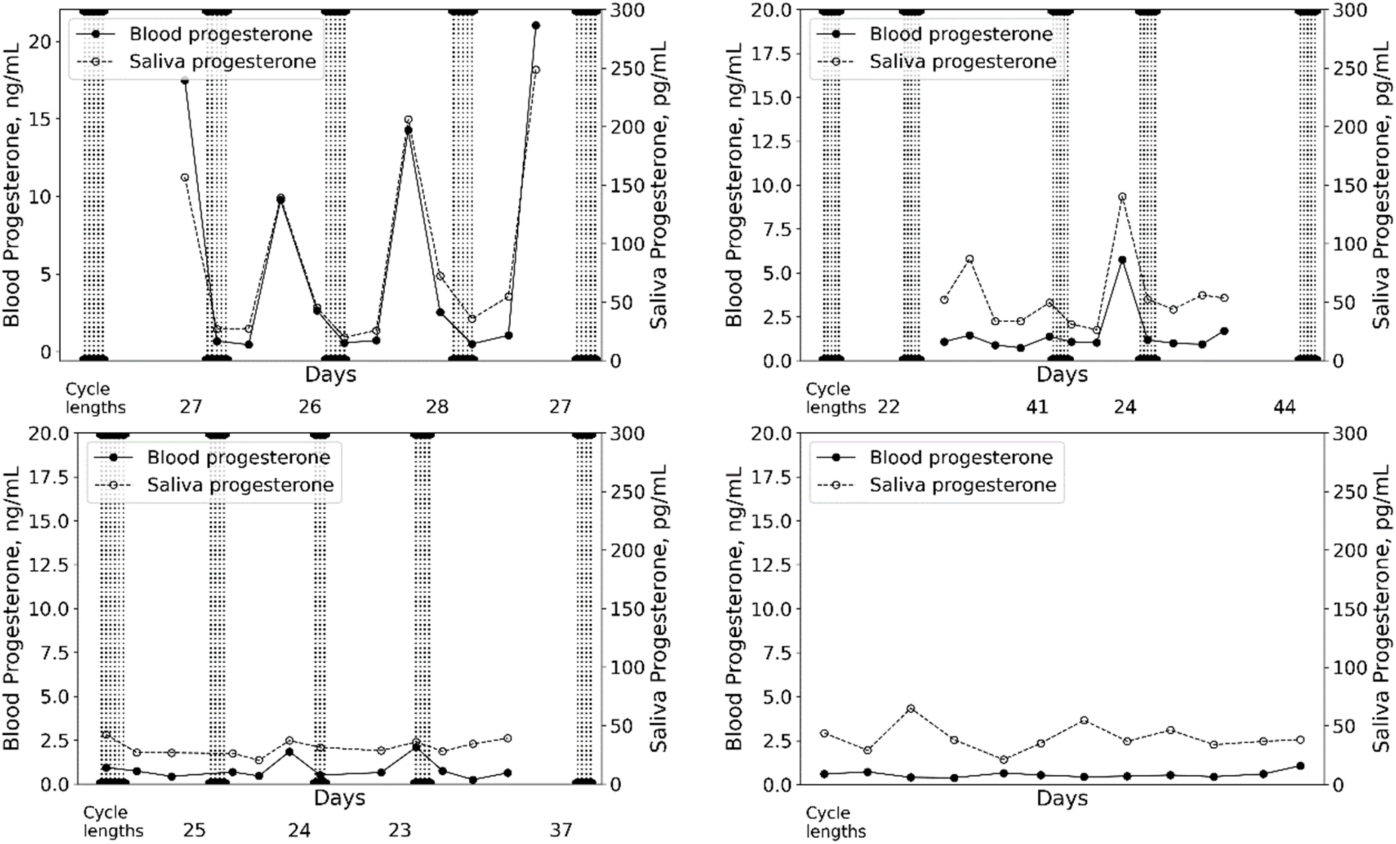
Examples of capillary blood and salivary progesterone measured across menstrual cycles in a eumenorrheic-ovulatory player **(A)** an oligomenorrheic player **(B)** an anovulatory player **(C)** and an amenorrheic player **(D)** vertical lines indicate days of menses.

The strength of the association between saliva and capillary blood appears to be concentration dependent. A strong and significant correlation was observed in the eumenorrheic group, which weakened with menstrual irregularities; a moderate significant correlation was found in the oligomenorrheic group but there were no significant correlations found in the anovulatory or amenorrhoeic groups (Figure 2). The weaker correlation observed in the individuals with menstrual irregularities may be due to the intra-individual range in hormonal concentration and a function of the reduced variability in both the saliva and capillary blood data i.e., the menstrual irregularities observed reduced the natural variability of progesterone concentration across each MC. Alternatively the lack of association could be due to the small sample size of this study.

### 4.2 Protocol efficacy

Menstrual cycle monitoring is increasingly utilised in research and applied practice to help inform the health and performance preparation of female athletes. Despite methodological guidance (4) many studies and day-to-day applied practice fail to quantitatively measure ovarian hormones, relying only on the presence of menses and counting days for MC length and phases. In our study, luteal phase progesterone concentrations of >2 ng/ml were consistently observed across the MCs in 10 out of the 21 players investigated and these cycles were classified as assumed ovulatory (20). We acknowledge that the gold-standard methodology to robustly characterise ovarian hormone profiles would include urinary ovulation kits to establish the mid-cycle luteinizing hormone surge and blood sampling in 4 phases of the MC (27, 28) but this study took place within the constraints of a professional football environment where availability of players, time and resource requirements often make research challenging. Therefore, this study represents an attempt to establish a protocol that was practically feasible in an elite football environment as well as ecologically valid. Despite only measuring progesterone once per week, we were still able to observe the known inter and intra-variation that exists in the concentrations and fluctuations of ovarian hormones in circulation (8, 29), and use that data to characterise a hormone profile of the cohort in a way that would not have been possible had we simply relied on the presence of menses and calendar based MC length to assess MC status. Indeed, of the 21 players we monitored, 11 had luteal phase progesterone concentrations <2 ng/ml, suggestive of a menstrual irregularity, despite the presence of regular menses (Table 1). If progesterone had not been measured, these players may have been falsely classified as ovulatory and may not have received potentially crucial further investigation.

Despite the practical benefits of sampling all of the players on the same day each week, irrespective of where each player was within their own MC, we do acknowledge that this may have resulted in missing the luteal rise in progesterone on some occasions and that more targeted and individualised sampling, with the possible addition of urinary ovulation testing, would be desirable, to more rigorously establish ovarian hormone profiles.

### 4.3 Saliva criteria

Reference ranges for serum progesterone are well established (20). Although saliva provides a more accessible means of measuring hormones in a research and applied setting there are currently no confirmed thresholds for salivary progesterone. We measured progesterone concentrations, weekly for 12 weeks in 21 elite football players. We identified that a luteal phase salivary progesterone level exceeding 50 pg/ml and being greater than 1.5 times the follicular baseline demonstrated good sensitivity (0.86), specificity (0.92), and accuracy (0.88) in indicating ovulation when compared to established criteria for blood progesterone (20) Using these criteria, we classified ten players as eumenorrheic, six as oligomenorrheic, four as anovulatory and one player as amenorrhoeic (Table 1).

## 5 Considerations and future directions

High variability in hormone concentration and MC irregularities are common in the first 2–3 years post menarche (30, 31). Menstrual cycles can often range between 21 and 45 days, with lower progesterone and high incidence of anovulatory cycles and oligomenorrhea (31, 32). Full gynaecological maturity can take up to 6 years post menarche (33). The players in our study ranged between 4 and 9 years post-menarche, so it is possible that the high proportion of oligomenorrhea and anovulatory cycles we observed was influenced by the gynaecological maturity of some of the group. The number of young players training in elite football environments is increasing rapidly (34), so future MC research should focus on larger cohorts of young female players and consider the influence of gynecological age, to help refine criteria for ovarian hormone profiling and help establish this efficient and non-invasive method of MC monitoring.

## 6 Conclusions

Salivary measurements of progesterone are well correlated with capillary blood when taken during assumed ovulatory MCs. The strength of the association appears to be concentration dependent. A luteal phase salivary P4 > 50 pg/ml and >1.5× follicular baseline has good sensitivity, specificity, and accuracy to indicate ovulation compared to established criteria for blood progesterone. Whilst this study has shown weekly measurements of salivary progesterone can estimate if ovulation has occurred, the criteria established here should be tested in larger studies, across a range of populations. Furthermore, research to help establish a “cut-off” for luteal phase salivary progesterone would also aid in establishing this efficient and non-invasive method of MC monitoring.

## Data Availability

The raw data supporting the conclusions of this article will be made available by the authors, without undue reservation.

## References

[1] De Souza MJ Toombs RJ Scheid JL O'Donnell E West SL Williams NI. High prevalence of subtle and severe menstrual disturbances in exercising women: confirmation using daily hormone measures. Hum. Reprod. (2010) 25(2):491–503. 10.1093/humrep/dep411 19945961

[2] Gimunová M Paulínyová A Bernaciková M Paludo AC. The prevalence of menstrual cycle disorders in female athletes from different sports disciplines: a rapid review. Int J Environ Res Public Health. (2022) 19(21):14243. 10.3390/ijerph192114243 36361122 PMC9658102

[3] Martínez-Fortuny N Alonso-Calvete A Da Cuña-Carrera I Abalo-Núñez R. Menstrual cycle and sport injuries: a systematic review. Int J Environ Res Public Health. (2023) 20(4):3264. 10.3390/ijerph20043264 36833966 PMC9958828

[4] Colenso-Semple LM D’Souza AC ElliottSale KJ Phillips SM. Current evidence shows no influence of women’s menstrual cycle phase on acute strength performance or adaptations to resistance exercise training. Front Sports Act. (2023) 5:1054542. 10.3389/fspor.2023.1054542 37033884 PMC10076834

[5] Dam TV Dalgaard LB Sevdalis V Bibby BM Janse DE Jonge X Muscle performance during the menstrual cycle correlates with psychological well-being, but not fluctuations in sex hormones. Med Sci Sports Exerc. (2022) 54(10):1678–89. 10.1249/MSS.0000000000002961 36106832 PMC9473716

[6] Legerlotz K Nobis T. Insights in the effect of fluctuating female hormones on injury risk-challenge and chance. Front Physiol. (2022) 13:827726. 10.3389/fphys.2022.827726 35250631 PMC8891628

[7] Dehghan F Soori R Yusof A. Knee laxities changes with sex-steroids throughout the menstrual cycle phases in athlete and non-athlete females. Rev Bras Ortop (Sao Paulo). (2024) 59(1):e29–37. 10.1055/s-0043-1771007 38524710 PMC10957278

[8] Shea AA Vitzthum VJ. The extent and causes of natural variation in menstrual cycles: integrating empirically based models of ovarian cycling into research on women’s health. Drug Discov Today Dis Models. (2020) 32:41–9. 10.1016/j.ddmod.2020.11.002

[9] Anckaert E Jank A Petzold J Rohsmann F Paris R Renggli M Extensive monitoring of the natural menstrual cycle using the serum biomarkers estradiol, luteinizing hormone, and progesterone. Pract Lab Med. (2021) 25:e00211. 10.1016/j.plabm.2021.e00211 33869706 PMC8042396

[10] Reed BG Carr BR. The Normal Menstrual Cycle and Control of Ovulation. Endotext, MDText.com. Feingold KR Anawalt B Blackman MR Boyce A Chrousos G Corpas E, editors. South Dartmouth, MA: MDText.com. Inc (2000). 25905282

[11] Stern J Arslan RC Penke L. Stability and validity of steroid hormones in hair and saliva across two ovulatory cycles. Compr Psychoneuroendocrinol. (2022) 9:100114. 10.1016/j.cpnec.2022.100114 35755924 PMC9216405

[12] Connor ML Sanford LM Howland BE. Saliva progesterone throughout the menstrual cycle and late pregnancy. Can J Physiol Pharmacol. (1982) 60(3):410–3. 10.1139/y82-060 7074426

[13] Gröschl M Rauh M Schmid P Dörr HG. Relationship between salivary progesterone, 17-hydroxyprogesterone, and cortisol levels throughout the normal menstrual cycle of healthy postmenarcheal girls. Fertil Steril. (2001) 76(3):615–7. 10.1016/s0015-0282(01)01960-4 11532491

[14] Vining RF McGinley RA Symons RG. Hormones in saliva: mode of entry and consequent implications for clinical interpretation. Clin Chem. (1983) 29(10):1752–6. PMID: . 6225566

[15] Gandara BK Leresche L Mancl L. Patterns of salivary estradiol and progesterone across the menstrual cycle. Ann N Y Acad Sci. (2007) 1098:446–50. 10.1196/annals.1384.022 17435149 PMC2096416

[16] Chatterton RT Jr Mateo ET Hou N Rademaker AW Acharya S Jordan VC Characteristics of salivary profiles of oestradiol and progesterone in premenopausal women. J Endocrinol. (2005) 186(1):77–84. 10.1677/joe.1.06025 16002538

[17] Papacosta E Nassis GP. Saliva as a tool for monitoring steroid, peptide and immune markers in sport and exercise science. J Sci Med Sport. (2011) 14(5):424–34. 10.1016/j.jsams.2011.03.004 21474377

[18] Macias-Muñoz L Filella X Augé JM Hanzu FA Morales-Ruiz M Bedini JL Performance evaluation of siemens atellica enhanced estradiol assay. Adv Lab Med. (2020) 1(1):20190009. 10.1515/almed-2019-0009 37362558 PMC10197438

[19] Sakkas D Howles CM Atkinson L Borini A Bosch E Bryce C A multi-centre international study of salivary hormone oestradiol and progesterone measurements in ART monitoring. Reprod Biomed Online. (2021) 42(2):421–8. 10.1016/j.rbmo.2020.10.012 33279419

[20] Wideman L Montgomery MM Levine BJ Beynnon BD Shultz SJ. Accuracy of calendar-based methods for assigning menstrual cycle phase in women. Sports Health. (2013) 5(2):143–9. 10.1177/1941738112469930 24427382 PMC3658377

[21] Pedregosa F Varoquaux G Gramfort A Michel V Thirion B Grisel O Scikit-learn: machine learning in python. J Mach Learn Res. (2011) 12(85):2825–30.

[22] Plymate SR Namkung PC Metej LA Petra PH. Direct effect of plasma sex hormone binding globulin (SHBG) on the metabolic clearance rate of 17-estradiol in the primate. J Steroid Biochem. (1990) 36. 10.1016/0022-4731(90)90223-F 2391961

[23] Wood P. Salivary steroid assays—research or routine? Ann Clin Biochem. (2009) 46(Pt 3):183–96. 10.1258/acb.2008.008208 19176642

[24] Wong YF Mao K Panesar NS Loong EPL Chang AMZ Mi ZJ. Salivary estradiol and progesterone during the normal ovulatory menstrual cycle in Chinese women. Eur J Obstet Gynecol Reprod Biol. (1990) 34(1–2):129–35. 10.1016/0028-2243(90)90016-t 2303146

[25] Fiers T Dielen C Somers S Kaufman JM Gerris J. Salivary estradiol as a surrogate marker for serum estradiol in assisted reproduction treatment. Clin Biochem. (2017) 50(3):145–9. 10.1016/j.clinbiochem.2016.09.016 27668549

[26] Dielen C Fiers T Somers S Deschepper E Gerris J. Correlation between saliva and serum concentrations of estradiol in women undergoing ovarian hyperstimulation with gonadotropins for IVF/ICSI. Facts Views Vis Obgyn. (2017) 9(2):85–91. PMID: . 29209484 PMC5707777

[27] Elliott-Sale KJ Minahan CL de Jonge XAKJ Ackerman KE Sipilä S Constantini NW Methodological considerations for studies in sport and exercise science with women as participants: a working guide for standards of practice for research on women. Sports Med. (2021) 51(5):843–61. 10.1007/s40279-021-01435-8 33725341 PMC8053180

[28] Schaumberg MA Jenkins DG Janse de Jonge XAK Emmerton LM Skinner TL. Three-step method for menstrual and oral contraceptive cycle verification. J Sci Med Sport. (2017) 20(11):965–9. 10.1016/j.jsams.2016.08.013 28684053

[29] Gann PH Giovanazzi S Van Horn L Branning A Chatterton RT Jr. Saliva as a medium for investigating intra- and interindividual differences in sex hormone levels in premenopausal women. Cancer Epidemiol Biomarkers Prev. (2001) 10(1):59–64. PMID: . 11205490

[30] Adams Hillard PJ. Menstruation in adolescents: what do we know? And what do we do with the information? J Pediatr Adolesc Gynecol. (2014) 27(6):309–19. 10.1016/j.jpag.2013.12.001 25438706

[31] Peña AS Doherty DA Atkinson HC Hickey M Norman RJ Hart R. The majority of irregular menstrual cycles in adolescence are ovulatory: results of a prospective study. Arch Dis Child. (2018) 103(3):235–9. 10.1136/archdischild-2017-312968 28794095

[32] Metcalf MG Skidmore DS Lowry GF Mackenzie JA. Incidence of ovulation in the years after the menarche. J Endocrinol. (1983) 97(2):213–9. 10.1677/joe.0.0970213 6854190

[33] Serret Montoya J Hernández Cabezza A Mendoza Rojas O Cárdenas Navarrete R. Alteraciones menstruales en adolescentes. Bol Med Hosp Infant Mex. (2012):63–76.

[34] FIFA. FIFA women’s football: member associations survey report. (2023). Available online at: https://digitalhub.fifa.com/m/28ed34bd888832a8/original/FIFA-Women-s-Football-MA-Survey-Report-2023.pdf (Accessed August 12, 2024).

